# Alfabetización estadística y comunicación de riesgo para la vacunación contra la COVID-19: una revisión de alcance

**DOI:** 10.26633/RPSP.2021.108

**Published:** 2021-08-31

**Authors:** Yasna K. Palmeiro-Silva, Caroline Weinstein-Oppenheimer, Carlos Felipe Henríquez-Roldán, Shrikant I. Bangdiwala

**Affiliations:** 1 Institute for Global Health, University College London Londres Reino Unido Institute for Global Health, University College London, Londres, Reino Unido.; 2 Centro de Investigación Farmacopea Chilena (CIFAR), Universidad de Valparaíso Valparaíso Chile Centro de Investigación Farmacopea Chilena (CIFAR), Universidad de Valparaíso, Valparaíso, Chile.; 3 Instituto de Estadística, Facultad de Ciencias, Universidad de Valparaíso Valparaíso Chile Instituto de Estadística, Facultad de Ciencias, Universidad de Valparaíso, Valparaíso, Chile.; 4 Department of Health Research Methods, Evidence, and Impact, McMaster University, Hamilton Ontario Canadá Department of Health Research Methods, Evidence, and Impact, McMaster University, Hamilton, Ontario, Canadá.

**Keywords:** Comunicación en salud, riesgo, infecciones por coronavirus, vacunas, alfabetización en salud, estadística, Health communication, risk, coronavirus infections, vaccines, health literacy, statistics, Comunicação em saúde, risco, infecções por coronavirus, vacinas, letramento em saúde, estatística

## Abstract

**Objetivo.:**

Describir el papel que desempeñan la alfabetización estadística y la correcta comunicación de riesgo en las estrategias de comunicación relacionadas con la vacunación contra la COVID-19.

**Métodos.:**

Se realizó una revisión de alcance en enero del 2021, con las palabras clave “*statistical literacy*”, “*risk communication”, “health communication*” y “*pandemic*” en las bases de datos de la Biblioteca Virtual en Salud de la Organización Panamericana de la Salud, PubMed, Web of Science, EBSCO y Google Académico. No se aplicaron filtros para fechas, idioma o tipos de publicación.

**Resultados.:**

De los 87 artículos identificados, cuatro cumplieron con los criterios de inclusión. Se reconocieron cuatro mensajes principales que relacionan la alfabetización estadística y la comunicación de riesgo: 1) la comunicación de riesgo y el nivel de alfabetización estadística afectan a la toma de decisión individual y colectiva, 2) la comunicación de la incertidumbre debe incluir lo que se sabe y no se sabe respecto a las estadísticas y los riesgos, 3) el uso de gráficos y visualizaciones es clave para informar apropiadamente a la población y 4) deben utilizarse diferentes formatos para mejorar la comunicación, siempre ajustados al nivel de alfabetización estadística de la población.

**Conclusiones.:**

La alfabetización estadística desempeña un papel clave en la comunicación de los riesgos relacionados con la salud en general y la vacunación contra la COVID-19 en particular. En situaciones de emergencia sanitaria, la correcta comunicación de riesgo y de la incertidumbre asociada debe ser clara, transparente y oportuna.

## Abstract (en)

**Objective.:**

To describe the role of statistical literacy and proper risk communication in communication strategies related to COVID-19 vaccination.

**Methods.:**

A scoping review was carried out in January 2021, with the keywords “statistical literacy,” “risk communication,” “health communication,” and “pandemic,” in the Pan American Health Organization Virtual Health Library, PubMed, Web of Science, EBSCO, and Google Scholar databases. No filters were applied for dates, language, or publication type.

**Results.:**

Of the 87 articles identified, four met the inclusion criteria. Four main messages were recognized that relate statistical literacy and risk communication: 1) risk communication and statistical literacy level affect individual and collective decision-making; 2) communication of uncertainty should include what is known and not known with regard to statistics and risks; 3) the use of graphics and visuals is key to appropriately informing the population; and 4) different formats should be used to improve communication, always adjusted to the population’s statistical literacy level.

**Conclusions.:**

Statistical literacy plays a key role in communicating risks related to health in general and COVID-19 vaccination in particular. In health emergencies, proper communication of risk and associated uncertainty should be clear, transparent, and timely.

## RESUMO

**Objetivo.:**

Descrever o papel da literacia estatística e da comunicação correta de riscos nas estratégias de comunicação relacionadas à vacinação contra a COVID-19.

**Métodos.:**

Em janeiro de 2021, foi realizada uma revisão de escopo com as palavras-chave “*statistical literacy*”, “*risk communication”, “health communication*” e “*pandemic*” nos bancos de dados da Biblioteca Virtual em Saúde da Organização Pan-Americana da Saúde, PubMed, Web of Science, EBSCO e Google Scholar. Não foi aplicado filtro de data, idioma ou tipo de publicação.

**Resultados.:**

Dos 87 artigos identificados, quatro atenderam aos critérios de inclusão. Foram reconhecidas quatro mensagens principais relacionadas à literacia estatística e à comunicação de risco: 1) a comunicação de risco e o nível de literacia estatística afetam a tomada de decisão individual e coletiva; 2) a comunicação da incerteza deve incluir o que é conhecido e desconhecido sobre estatísticas e riscos; 3) o uso de gráficos e visualizações é fundamental para informar adequadamente a população; e 4) devem ser usados diferentes formatos para melhorar a comunicação, sempre ajustados ao nível de literacia estatística da população.

**Conclusões.:**

A literacia estatística desempenha um papel fundamental na comunicação dos riscos relacionados à saúde em geral e à vacinação contra a COVID-19 em particular. Em situações de emergência de saúde, a comunicação correta do risco e da incerteza a ele associada deve ser clara, transparente e oportuna.

El 30 de enero del 2020, la Organización Mundial de la Salud (OMS) declaró el virus SARS-CoV-2 como una emergencia sanitaria de interés internacional (1). Un año después, la discusión pasó a centrarse en las vacunas y los programas de vacunación contra la COVID-19, que comenzaron a finales del año 2020. Nunca antes se había visto un avance tan veloz en el desarrollo de una vacuna, desde las fases preclínicas hasta la aplicación masiva. Sin embargo, aún quedan grandes desafíos, que van desde la comunicación de la información esencial hasta la logística de la vacunación y el mantenimiento de las medidas sanitarias y de distanciamiento después de la inmunización.

La aversión a la incertidumbre, las preocupaciones de la población ante las medidas de contención del virus (cuarentenas y distanciamiento social) y los nuevos programas masivos de vacunación son elementos que incentivan tanto el interés de la población en la ciencia como la confianza en ella de las autoridades y tomadores de decisiones. Todo esto acentúa la importancia de lograr una adecuada comunicación de los riesgos relacionados con la salud, así como de la toma de decisiones informadas y bien fundamentadas que permitan contener la propagación del virus (2).

La comunicación de riesgo^5^ es esencial en salud pública, especialmente durante las emergencias sanitarias. La OMS recomienda que la comunicación de riesgo sea transparente, oportuna, fácil de entender, que reconozca la incertidumbre en la información y que se divulgue a través de diferentes plataformas. Estas recomendaciones son amplias, pero resultan poco concretas para los tomadores de decisiones y las autoridades encargadas de comunicar el riesgo (3). Por ello, la OMS y las Naciones Unidas publicaron una guía para la recuperación pos-COVID-19, en la que se destaca la necesidad de fortalecer los canales de comunicación e incluir las tecnologías digitales (4). No obstante, las recomendaciones continúan siendo generales y en ellas no se considera la importancia de la alfabetización estadística y su influencia positiva en la comprensión y el uso de la información para la correcta toma de decisiones por parte de las autoridades sanitarias y la población general.

Se entiende por alfabetización estadística la habilidad para leer, interpretar, evaluar críticamente, discutir, y comunicar la información estadística y su significado (5, 6). Así, un bajo nivel de alfabetización estadística llevaría a un inadecuado entendimiento, procesamiento y uso de la información estadística relacionada con la salud —lo que podría llevar a tomar decisiones inadecuadas que podrían influir negativamente en el nivel de protección de la salud individual y colectiva—; además, favorecería la diseminación de información errónea (para lo que se ha acuñado el término en inglés *misinformation)* o falsa (*fake news*) que reducen la efectividad de las medidas sanitarias y pueden incitar a prácticas riesgosas. La alfabetización en salud —un concepto más cercano a la población— también desempeña un papel clave en la toma de decisiones correctas y en la adhesión de la población a las medidas sanitarias recomendadas (7, 8).

Al combinar los elementos de comunicación de riesgo durante una emergencia sanitaria y la alfabetización estadística, se deben tomar en cuenta los elementos básicos de toda comunicación —emisor, mensaje y receptor— y el nivel de alfabetización estadística de la población. Como sería poco factible elevar el nivel de alfabetización estadística del receptor durante una pandemia mediante entrenamientos formales —debido a la urgencia de la situación—, lo aconsejable es fortalecer el nivel de alfabetización estadística del emisor y la claridad del mensaje. En otras palabras: el emisor debe comprender de manera adecuada lo que desea transmitir, para luego entregar el mensaje de manera clara, simple, informativa y precisa.

En este estudio se describe el papel que desempeñan la alfabetización estadística y la correcta comunicación de riesgo en las estrategias de comunicación relacionadas con la vacunación contra la COVID-19.

## MATERIALES Y MÉTODOS

Se realizó una revisión de alcance ajustada a la metodología propuesta por Arksey y O'Malley (9) y las recomendaciones de PRISMA-ScR (10). La pregunta de investigación que guio la búsqueda fue “¿*Cuál es el papel y la importancia de la alfabetización estadística en la comunicación de riesgo durante una pandemia*?”

Para la búsqueda se emplearon combinaciones de los siguientes términos en inglés: “*statistical literacy*”, “*risk communication”, “health communication*” y “*pandemic*”. Se consultaron las bases de datos de la Biblioteca Virtual en Salud de la Organización Panamericana de la Salud, PubMed, Web of Science, EBSCO y Google Académico. No se aplicaron filtros para fechas, idioma o tipos de publicación. La búsqueda se realizó el 31 de enero del 2021.

Uno de los autores del estudio realizó la búsqueda y seleccionó los artículos según los criterios de elegibilidad: publicaciones en inglés, español o portugués que abordaran la alfabetización estadística y la comunicación de riesgo de manera relevante para la pregunta de investigación. Una vez eliminados los duplicados, la selección de artículos se realizó en tres etapas. Primero, se evaluaron los títulos de los artículos, luego los resúmenes y finalmente los textos completos. Dado que el objetivo de esta revisión era evaluar el alcance y la amplitud de toda la evidencia disponible, no se realizó el análisis crítico de los materiales seleccionados.

Este estudio no requirió de aprobación por parte de un comité de ética, ya que solo analizó información públicamente disponible y no involucró el manejo de información personal.

## RESULTADOS

**FIGURA 1.**
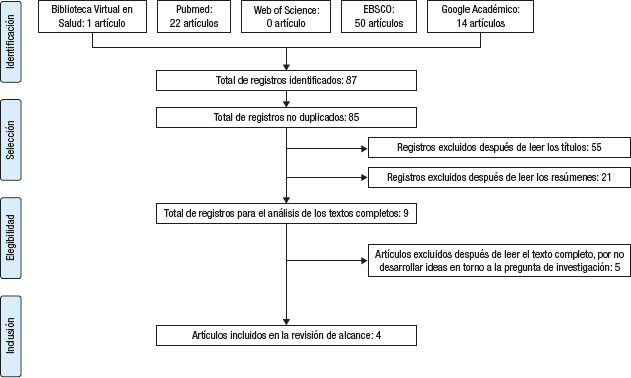
Resultados de la selección de los artículos, según las etapas del proceso

Se identificaron 87 artículos. Después de eliminar los duplicados y de aplicar los criterios de inclusión, se seleccionaron cuatro artículos para el análisis de contenido (figura 1); de ellos, tres eran artículos de comentario o perspectiva (11–13) y el otro era el capítulo de un libro (14).

A partir de los mensajes clave de los artículos analizados (cuadro 1), se agrupó la información en cuatro tópicos que relacionan la alfabetización estadística y la comunicación de riesgo:

1. *Impacto de la comunicación de riesgo en la toma de decisiones individual y poblacional*. En la comunicación de riesgo, se debe ofrecer la información de manera comprensible, no sesgada y completa, poniendo en su centro a la población, para que esta pueda tomar decisiones de manera informada. Un bajo nivel de alfabetización estadística reduce las posibilidades de tomar decisiones correctas (14).
2. *Comunicación de la incertidumbre.* Es clave la comunicación clara y precisa de: a) las incertidumbres respecto a lo que se sabe y no se sabe en las estadísticas sobre la COVID-19; b) la seguridad, la eficacia y la efectividad de las vacunas, y c) el grado de consenso o no de las opiniones de los expertos. La insuficiente comprensión de las incertidumbres puede deberse a un bajo nivel de alfabetización estadística o a sesgos cognitivos en la población (12, 13); esto, a su vez, puede debilitar la percepción del riesgo y reducir la confianza en las autoridades (11).
3. *Uso de gráficos y visualizaciones.* El uso de gráficos y visualizaciones es clave para informar apropiadamente a la población y deben analizarse cuidadosamente, ya que no todos los formatos son fácilmente interpretables. Los pictogramas pueden ser apropiados para informar el riesgo de una enfermedad, pero si la intención es mostrar el grado de incertidumbre respecto a la proyección de la tasa de esa enfermedad, sería mejor usar un gráfico que incorpore los intervalos de incertidumbre (12, 13). A su vez, la información estadística debe ser explicada en formatos numéricos y descriptivos, por ejemplo mediante categorías (13).
4. *Elementos para mejorar la comunicación cuando el nivel de alfabetización en la población es bajo.* Los formatos utilizados para transmitir el mensaje pueden influir en las reacciones emocionales y cognitivas del receptor; por lo tanto, estos formatos deben considerar el nivel de alfabetización estadística de la audiencia (14). Los mensajes hablados deben transmitir empatía y respeto por el público, y los debe comunicar una persona reconocida como experto o experta, y que goce de la confianza de la población (11).

***Fuente:*** elaborado por los autores.

**CUADRO 1.** Síntesis de los artículos incluidos en la revisión

Artículo	Mensajes clave
Paek y Hove (11)	- Hay dos tipos de incertidumbres: la epistémica y la de consenso. - Inciden dos factores en la comunicación de riesgo: la importancia de un correcto formato en los mensajes, y la experticia y confianza de la persona que entrega el mensaje. - Es clave explicar las discrepancias y las incertidumbres de la información.
Groth (12)	- Las autoridades y profesionales de la salud deben dominar el llamado conocimiento estadístico para la educación, es decir, deben comprender los conceptos estadísticos para poder comunicar claramente la información estadística a la poblacion. - Se deben reconocer la incertidumbre y los diversos procesos estocásticos, más que determinísticos, que influyen en la salud.
Bhatt et al. (13)	- Existen dos tipos de incertidumbres: la aleatoria y la epistémica. - Hay dos tipos de métodos para cuantificar la incertidumbre: los bayesianos y los que se basan en la frecuencia. - La comunicación de las incertidumbres puede percibirse imprecisa por: 1) diferentes niveles de comprensión de las probabilidades y la estadística; 2) sesgos personales que podrían influir en la comprensión de la información. - Es clave seleccionar los métodos apropiados de comunicación.
Bodemer y Gaissmaier (14)	- Existen dos modos de comunicar: el persuasivo y el informativo; este último considera que el receptor es capaz de tomar decisiones informadas según los riesgos y las incertidumbres. - Las personas con buen nivel de alfabetización estadística toman mejores decisiones para su salud. - Los gráficos pueden ayudar a las personas con bajo nivel de alfabetización estadística; revelar las incertidumbres puede ayudar a evitar la ilusión de certidumbre.

***Fuente:*** elaborado por los autores.

## DISCUSIÓN

Los resultados de esta revisión de alcance reflejan la limitada evidencia científica que enlaza la alfabetización estadística y la comunicación de riesgo; este vacío podría influir negativamente en la comprensión cabal y el uso apropiado de estos procesos vitales para la salud pública. A pesar de que en la búsqueda se utilizó la palabra clave “pandemia” con el objetivo de ampliar la búsqueda y no limitarla a la actual pandemia de COVID-19, los resultados fueron escasos.

Este hallazgo es relevante, ya que la alfabetización estadística desempeña un papel clave en la comunicación de riesgo y la salud pública en general, especialmente en el momento actual por la pandemia de la COVID-19, ya sea porque las tasas de contagio y de mortalidad continúan elevadas en varios países del mundo o por los desafíos asociados a los procesos de vacunación, incluida la reticencia a vacunarse. Metzger y colaboradores encontraron que las personas con mayor nivel de alfabetización estadística entienden el riesgo de infección por la COVID-19 de manera más precisa y cumplen mejor las medidas de distanciamiento social y la vacunación (15).

Aunque a un año y medio del inicio de la pandemia, los tomadores de decisiones y la población ya estaban más familiarizados con las medidas para la contención del virus SARS-CoV-2, los desafíos para la salud pública continúan presentes, tanto a nivel global como nacional.

A partir del análisis de la evidencia recabada, se puede llegar a algunas precisiones y recomendaciones relevantes a la hora de considerar la comunicación de riesgo, la alfabetización estadística y los procesos de vacunación.

## Impacto de la comunicación de riesgo en la toma de decisiones a nivel individual y poblacional

La comunicación de los riesgos relacionados con la salud debe basarse en mensajes claros para coadyuvar a una mejor y más correcta toma de decisiones por parte de la población. Con relación a las vacunas y tomando como base las cifras de efectividad, se destacan los siguientes dos puntos clave en la comunicación de riesgo.

**Eficacia *vs*. efectividad.** El desarrollo de una vacuna incluye varias fases de ensayos clínicos: una vez producida a nivel de laboratorio y probada en animales (etapa preclínica), se debe comprobar su seguridad en voluntarios humanos para evaluar posibles efectos adversos (fase I) y su inmunogenicidad (fase II), hasta llegar a los ensayos de eficacia (fase III). En la fase III se evalúa el desempeño de la vacuna bajo circunstancias controladas (16), es decir, se comparan las tasas de incidencia de una enfermedad transmisible en personas vacunadas y no vacunadas con similar exposición al virus. Una vez comprobadas la seguridad, la inmunogenicidad y la eficacia de la vacuna, se puede medir la efectividad, definida como el desempeño de la vacuna en circunstancias reales (16), es decir, sin controlar las variables que pudiesen alterar el efecto de la vacuna. Si bien el personal de salud y los tomadores de decisiones en el área de la salud suelen estar familiarizados con estos términos, no ocurre así con la población general. La resistencia a la vacunación por parte de algunos grupos podría estar influenciada por la falta de conocimiento de los procesos de desarrollo y validación de la vacuna y por “malos entendidos” de la jerga científica respecto a los ensayos clínicos y las cifras derivadas de ellos (17–19). Para poder informar correctamente a la población, estos elementos deben ser parte de la comunicación de riesgo.

Por ejemplo, en la publicación científica de Polack y colaboradores sobre la eficacia de la vacuna desarrollada por Pfizer, el riesgo de la ocurrencia de COVID-19 al menos siete días después de la segunda dosis en participantes sin evidencia de infección previa se presenta de tres maneras: 1) eficacia (95,0%); 2) intervalo de credibilidad, según la estadistica bayesiana, (90,3% a 97,6%) y 3) probabilidad de que la eficacia sea más del 30% (>99,99%) (20). La forma en que estas cifras han sido presentadas en la prensa resalta el porcentaje de eficacia: “*La primera dosis de la vacuna comienza a proteger a los pacientes diez días después de la inyección, y la segunda dosis reduce el riesgo de desarrollar la COVID-19 en 95%, en comparación con no estar vacunado.”* (21). En ese reportaje se interpreta correctamente el 95,0% como la reducción en el riesgo; es decir, si el riesgo de desarrollar la COVID-19 fuera *X*% sin vacuna, el riesgo en los vacunados sería (1 - 0,95)**·***X*, expresado en porciento. Sin embargo, para captar el sentido de ese riesgo relativo, se debe presentar también el riesgo absoluto de desarrollar la COVID-19, que refleja la probabilidad de enfermar en condiciones específicas: en ese estudio, el riesgo de desarrollar la COVID-19 en los no vacunados fue de 0,925% y en los vacunados de 0,046% [producto de la ecuación (1 - 0,95)**·**0,925%)] (20); es decir, el riesgo de desarrollar la COVID-19 sin vacuna según ese estudio fue ligeramente menor de 1%. Una cosa es el **riesgo absoluto** de desarrollar la COVID-19 (0,046%) estando vacunados y otra diferente es el **riesgo relativo** de desarrollar la COVID-19 (5,0%) estando vacunados.

**Diferentes tipos de vacunas contra la COVID-19.** Otra fuente de confusión es la existencia de diferentes plataformas tecnológicas para desarrollar las vacunas y la necesidad de comparar las eficacias resultantes de los estudios clínicos efectuados con principios activos y diseños experimentales diferentes. Entre las vacunas que cuentan con la aprobación para uso de emergencia emitida por alguna agencia regulatoria nacional en el mundo se encuentran la BNT162b (Pfizer-BioNTech) y la mRN-1273 (Moderna), con valores de eficacia de 95,0% (20) y 94,1% (22), respectivamente. Ambas vacunas se basan en ácido ribonucleico mensajero (ARNm) modificado que codifica para la proteína S, clave para el ingreso del virus a la célula; se debe resaltar que esta plataforma se utilizó —y aprobó— para vacunas por primera vez en el mundo (23). También se aprobaron para uso de emergencia vacunas basadas en el virus inactivado químicamente, como la CoronaVac (Sinovac) y la BBIBP-CorV (Sinopharm), con eficacias de 50,4% (24) y 79,3% (25), respectivamente. Finalmente, una de las plataformas más estudiadas es la basada en vectores adenovirales que portan el código de ácido desoxirribonucleico (ADN) para producir la proteína S en el organismo del vacunado, como la ChAdOx1 COVID-19 (Oxford–AstraZeneca), la Gam-COVID-Vac (Sputnik V) y la Ad26.COV2.S (Janssen), que mostraron eficacias de 66,7%, 91,6% y 66,9%, respectivamente (26, 27). Otra vacuna perteneciente a este grupo es la Ad5-nCoV (Convidecia o CanSino), pero al momento de escribir este artículo no se había verificado su eficacia^6^. Es importante resaltar que, si bien los perfiles de seguridad de todas estas vacunas son semejantes en términos generales, no todas ellas demostraron la misma eficacia en diferentes grupos de edad. La correcta interpretación y comunicación de esta información resulta difícil debido a los diferentes diseños de estudio y poblaciones incluidas en ellos, por lo que se debe tener especial cuidado de no generalizar inadecuadamente los resultados.

## Comunicación de la incertidumbre y los riesgos

La Asociación Internacional para la Educación Estadística promueve la alfabetización estadística en todo el mundo, de manera que la población pueda comprender la información especializada e interpretar correctamente los datos de incertidumbre, propios de la investigación científica (28). La incertidumbre es uno de los conceptos fundamentales de la estadística y proviene de dos fuentes: 1) el hecho de que todo proceso —biológico, social, económico, ambiental, etc.— es variable y 2) casi nunca observamos todas las ocurrencias de un proceso, sino solo una muestra o parte de él. Aunque eso genera una determinada incertidumbre que no se expresa en términos de certezas sino de probabilidades, sin una adecuada alfabetización estadística, el público en general entenderá la información en términos de certezas. De hecho, la población espera que una vacuna prevenga la COVID-19 en 100%, por lo que al ver un valor de eficacia de 95,0% puede asumir que eso equivale al 100% y no considera el 5,0% de no eficacia. Incluso si se asume que el 95,0% es el valor verdadero de eficacia, no se toma en cuenta que esta cifra es solo un estimado y que, incluso con un alto nivel de confianza, puede oscilar entre 90,3% y 97,6%. Como las probabilidades no se aplican a un individuo en particular sino a una población, aunque una persona pueda concluir que una eficacia de 95,0% significa que, como individuo, no va a contraer la COVID-19, en realidad esa cifra indica que de 100 000 adultos vacunados en su comunidad, entre 2 400 y 9 700 podrían desarrollar la enfermedad. Estos detalles son muy importantes en la comunicación de riesgo, ya que, independientemente de lo que la población pueda creer sobre la eficacia de la vacuna, el mensaje tiene que ser claro y transparente, e incluir las posibles incertidumbres.

Por otra parte, se debe recordar que la eficacia de una vacuna es relativa. Existe un riesgo si una persona no se vacuna y la vacunación solo disminuye ese riesgo. Por eso se debe insistir en que hay otras formas de reducir el riesgo de desarrollar la enfermedad, como las medidas sanitarias y sociales —usar mascarillas apropiadamente, lavarse las manos, mantener distancia física al socializar— y el confinamiento. Estas medidas potencian el efecto de la vacunación en la población, por lo que se deben considerar al momento de evaluar los riesgos.

## Uso de gráficos y visualizaciones para apoyar la comunicación

**FIGURA 2.**
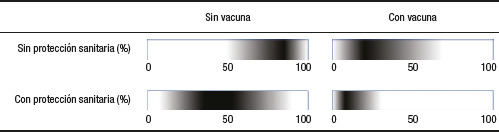
Ejemplo hipotético de cómo mostrar el estimado porcentual del riesgo de contraer la COVID-19 y la incertidumbre asociada^a^ bajo diferentes situaciones

Un desafío importante es cómo comunicar de manera simple pero precisa la información —incluidas las cifras estimadas y la incertidumbre— a un público que no solo no piensa en términos estadísticos, sino que incluso puede tener “miedo” a los números. Al informar sobre la COVID-19 y las vacunas, además de presentar las cifras, se debe incluir una narrativa de esas cifras que impida interpretaciones erróneas. Es altamente recomendable que esta información se presente mediante gráficos claros, por ejemplo infografías. Si se quiere mostrar la incertidumbre asociada a los estimados de riesgo bajo diferentes circunstancias, como en el ejemplo presentado en la figura 2, en vez de presentar números, se puede presentar una barra con valores que van de 0% a 100%, en la que el color más intenso se centra en el estimado puntual del riesgo más probable, rodeado de zonas grises que reflejan el intervalo de confianza de ese estimado y se hacen más claras hasta llegar al blanco (que indicaría que el riesgo indicado en esa zona ya no sería el correcto). En ese gráfico, la posición del color intenso muestra los estimados del riesgo de mayor confianza, y la amplitud del color muestra la incertidumbre en ese estimado.

En esa situación hipotética ilustrada en la figura 2, sin vacuna y sin protección sanitaria, el riesgo de contraer la COVID-19 es alto, disminuye con la protección sanitaria, disminuye acentuadamente con la vacuna y alcanza la máxima protección (menor riesgo) con la vacuna y medidas de protección sanitaria. Obviamente, los gráficos que se presenten dependerán de la vacuna en cuestión, el porcentaje de vacunados y la exposición al virus en la población analizada.

El uso de herramientas visuales al presentar la información puede facilitar la comprensión de los riesgos y hacer notar la incertidumbre en los estimados. Obviamente, en la medida en que los riesgos cambian en el tiempo, los gráficos deben cambiar.

## Elementos para mejorar la comunicación

Las autoridades sanitarias deben incluir entre sus funciones el fortalecimiento de la alfabetización estadística y sanitaria de la población, y promover el pensamiento crítico mediante preguntas que fomenten la reflexión ante la nueva información (29):

- ¿Cuán confiable son los números? Esto incluye ¿cuán rigurosamente se realizó el estudio?, ¿cuáles son los intervalos de incertidumbre o confianza del estudio?, ¿los resultados avalan la conclusión?
- ¿Cuán confiable es la fuente de información? En este punto se debe considerar ¿cuáles son los sesgos y los conflictos de intereses de la fuente de información?, ¿cómo se ha presentado la información (positiva o negativamente)?, ¿se han exagerado los títulos y encabezados de los informes?, ¿qué parte de la información podría no haberse presentado?
- ¿Cuán confiable es la interpretación de los resultados?, ¿en qué medida se ajusta la información al contexto y a otros datos presentados con anterioridad?, ¿es la información generalizable a toda la población o solo a una parte de ella?, ¿cuál es la magnitud del efecto del estudio presentado, demasiado bueno como para ser verdadero?

***Fuente:*** elaborado por los autores.

a El color más intenso se centra en el estimado porcentual del riesgo con mayor certidumbre y su intervalo de confianza de 95%; el color se aclara a medida que se reduce la certidumbre de los estimados del riesgo.

Además, en la comunicación de riesgo, las autoridades deben reconocer la existencia de varios riesgos diferentes que se deben comunicar al público, por ejemplo:

- el riesgo de ser infectado, pero no desarrollar la COVID-19
- el riesgo de ser infectado, desarrollar la COVID-19, pero solo con síntomas leves
- el riesgo de ser infectado, desarrollar la COVID-19 con síntomas moderados, pero sin necesidad de hospitalización
- el riesgo de ser infectado, desarrollar la COVID-19 y ser hospitalizado en una unidad de cuidados intensivos (UCI)
- el riesgo de ser infectado, desarrollar la COVID-19, ser hospitalizado en una UCI, pero sin intubación ni conexión a ventilación mecánica
- el riesgo de ser infectado, desarrollar la COVID-19 y ser hospitalizado en una UCI con intubación y conexión a ventilación mecánica
- el riesgo de ser infectado, desarrollar la COVID-19 y morir.

Al analizar esta propuesta, se debe tener en cuenta que esta se deriva de muy pocos estudios, la mayoría de ellos sin un análisis crítico y con niveles de calidad muy disímiles, que no fue evaluado. No obstante, se incluyó toda la evidencia publicada sobre el tema.

Se puede afirmar, por lo tanto, que la alfabetización estadística desempeña un papel clave en la comunicación de los riesgos relacionados con la salud en general y la vacunación contra la COVID-19 en particular. En situaciones de emergencia sanitaria, la correcta comunicación de riesgo y de la incertidumbre asociada debe ser clara, transparente y oportuna.

## Declaración.

Las opiniones expresadas en este artículo son responsabilidad de los autores y no reflejan necesariamente los criterios ni la política de la *Revista Panamericana de Salud Pública / Pan American Journal of Public Health* y/o de la Organización Panamericana de la Salud.

## Footnotes

5 La Organización Panamericana de la Salud define la comunicación de riesgo como el intercambio en tiempo real de información, recomendaciones y opiniones entre expertos o funcionarios, y personas que se enfrentan a una amenaza (riesgo) para su sobrevivencia, su salud, o su bienestar económico o social (ver más en https://www3.paho.org/hq/index.php?option=com_content&view=article&id=11400:la-comunicacion-de-riesgo-preguntas-frecuentes&Itemid=41610&lang=en).

6 En el momento de revisar esta publicación, la autoridad regulatoria cubana CECMED había aprobado la vacuna Abdala, basada en la proteína S recombinante y con una eficacia de 92,28%, para uso de emergencia (https://www.cecmed.cu/noticias/aprueba-cecmed-autorizo-uso-emergencia-candidato-vacunal-cubano-abdala).
